# Multiple Types of Developmental Dyslexias in a Shallow Orthography: Principles for Diagnostic Screening in Italian

**DOI:** 10.3390/brainsci14080743

**Published:** 2024-07-25

**Authors:** Daniela Traficante, Claudio Luzzatti, Naama Friedmann

**Affiliations:** 1 Department of Psychology, Catholic University of the Sacred Heart, 20123 Milan, Italy; 2 Scientific Institute, IRCCS E. Medea—Bosisio Parini, 23842 Lecco, Italy; 3 NeuroMI—Milan Center for Neuroscience, 20126 Milan, Italy; 4 Department of Psychology, University of Milano-Bicocca, 20126 Milan, Italy; claudio.luzzatti@unimib.it; 5 Language and Brain Lab, School of Education and Sagol School of Neuroscience, Tel Aviv University, Tel Aviv-Yafo 6997801, Israel; naamafr@tauex.tau.ac.il

**Keywords:** reading models, types of developmental dyslexias, reading error coding

## Abstract

A new dyslexia screening test for Italian, *Tiltan-IT*, is presented. The test was developed based on an integrated dual-route model of reading, which describes in detail specific mechanisms underpinning early visual processes as well as the lexical and the sublexical routes. The principle according to which the test was developed is that each dyslexia type is manifested in different kinds of errors and in different kinds of stimuli, and we therefore included stimuli sensitive to each dyslexia type in the test. *Tiltan-IT* is a reading aloud test that includes word, nonword, and word pair lists. The test was administered to 618 Italian-speaking children (2nd–8th grade). Each error produced by the children was classified through the coding scheme developed to detect the different types of dyslexias described by the reading model. The *Tiltan-IT* was able to identify 110 children with dyslexia. The identified dyslexia types included letter position dyslexia, attentional dyslexia, letter identity dyslexia, surface dyslexia, vowel dyslexia, consonant conversion dyslexia, multi-letter phonological dyslexia, voicing dyslexia. The results confirm that the selection of items in the *Tiltan-IT* enabled the detection of the wide variety of dyslexias in Italian, some of them for the first time, adding evidence for the cross-linguistic validity of multiple types of developmental dyslexias and for the dual-route model of reading.

## 1. Introduction

Reading is a multi-staged process, leading from written words to their meaning and pronunciation. Each of the stages of this process may be affected by a developmental or acquired deficit, giving rise to a different type of dyslexia [1,2]. In this study, we developed a screening test for Italian that allows for the identification of the various types of dyslexias. The development of this test was based on two main principles: (1) each dyslexia type affects different types of stimuli (e.g., long words, nonwords, irregular words), and therefore, the test includes stimuli that are sensitive to each of the dyslexias; (2) each type of dyslexia is manifested in different types of errors, and therefore, the analysis of the test includes not only the number of errors, but also the type of errors that each reader makes [3]. 

**Figure 1:**
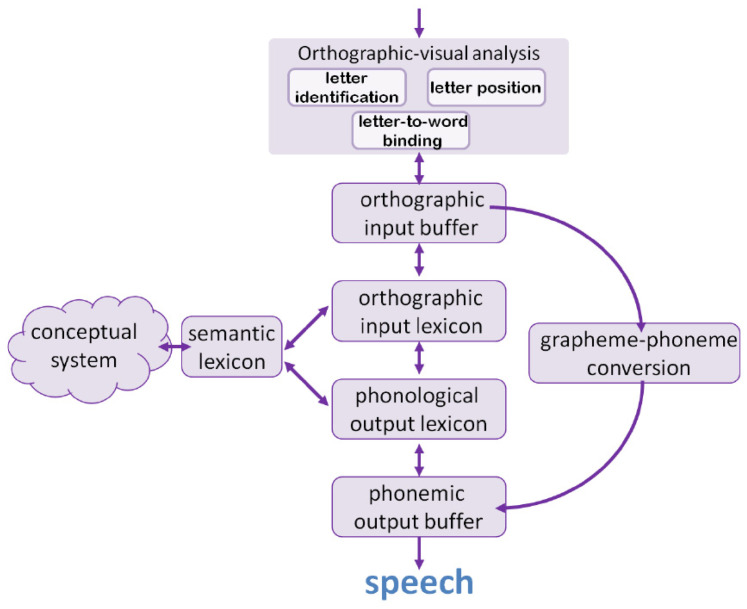
The dual-route model for single-word reading [1].

Models that describe the word and nonword reading process and place different developmental dyslexias along this process exist since the seminal work of John Marshall [4], and the models became more and more detailed through the deeper understanding of different types of dyslexias and from findings from typical readers. The current model we adopt here is the one developed on the basis of the dual-route model, proposed by Coltheart and his colleagues [2] (Dual-Route Cascaded—DRC—model) and by other research groups [5,6]. An updated version of this model, based on a wide range of studies on both acquired and developmental dyslexias, is described by Friedmann and Coltheart [1] (Figure 1). 

This model describes the specific mechanisms underpinning early orthographic–visual processes and the two routes in which the information further flows: a lexical and a sublexical one. It also describes the later stages of phonological output, shared by the lexical and the sublexical routes. The lexical route is used to read words that exist in the lexicons and is the only route that allows the correct reading of irregular and unpredictable words (e.g., PINT). It is characterized by the activation of whole-word representations in the orthographic input lexicon, which are connected to whole-word representations in the phonological output lexicon. The sublexical route allows the reading aloud of nonwords (e.g., RINT) and low-frequency regular words; through the application of grapheme-to-phoneme conversion (GPC) rules, it converts sequences of graphemes into sequences of phonemes, according to statistical rules set on distributional features of the language. 

The first component of reading aloud, according to this model, is the orthographic–visual analyzer, aimed at the early analysis of printed letter strings. Three mechanisms were described by several studies [7,8,9,10,11,12] as taking place in the early stages of letter-string perception: letter identification, letter position encoding within words, and letter-to-word binding. There is cross-linguistic evidence that each of these mechanisms may be selectively impaired, leading to different types of dyslexias. 

In the first step, the recognition of the abstract identity of an orthographic character (e.g., “A”) from the perception of the constituent tracts (e.g., /, \, -) is processed through the visual system. This mechanism allows a reader to identify letters, irrespective of the font or the case in which it is written (e.g., A, a, a). There is only one detailed case study of developmental dyslexia affecting this mechanism, i.e., a boy with a specific difficulty in identifying letters in isolation, who produced a high percentage of letter substitutions and omissions within words and nonwords and “don’t know” responses [13]. 

The specific developmental impairment in encoding letter position within words has already been described in many cases, in various languages. Friedmann and colleagues described the performance of Hebrew-speaking children [14,15,16,17,18,19], and the same profile of reading impairment was observed also in Arabic [20,21], in English [22,23,24], in Turkish [25], and in French [26]. Such a reading profile is called Letter Position Dyslexia (LPD) [15]. All individuals with LPD described in those studies produced a high percentage of reading errors in which they generated an anagram of the target word (e.g., SKATES read as “stakes”, FROM read as “form”) due to the migration of letters within words, mainly of middle letters. Far more migration errors occur in this dyslexia in migratable words, i.e., words in which a letter migration creates another existing word. Therefore, the probability of identifying LPD depends on the number of migratable words in a language: in languages like Hebrew that contain many migratable words, this dyslexia is evident even in random word lists; in languages with fewer migratable words, this dyslexia can be identified only when migratable words are selected and presented. Additionally, when migratable words appear within a meaningful text, the probability of migration errors is low because the reader may use context to constrain their errors; therefore, in reading texts, LPD is harder to detect (even though reading texts is still effortful for an individual with LPD). Therefore, the best stimuli to detect LPD in a screening test are migratable words presented in a list, as they are presented in the *Tiltan-IT*. 

A different type of migration error, migration between words, was originally described as Attentional Dyslexia (AD) by Shallice and Warrington [27] in two adults with acquired dyslexia, who did not make errors when reading words in isolation but produced migration errors between neighboring words, when words were presented among other words or letters (e.g., FIG TREE read as “fig free”). In this case, both letter identity (‘f’) and letter position coding are preserved (1st position), but letter-to-word binding is impaired. Letter migration between words can occur both horizontally and vertically and was observed in several cases of developmental dyslexia in Hebrew, Arabic, English, and French [21,26,28,29]. Beyond letter substitutions (e.g., LIGHT FATE read as “fight late”), the addition of a letter from a neighboring word (e.g., LIGHT FATE read as “flight late”) and the omission of a letter that appears in the two words in the same position (e.g., CLAY PLAN read as “clay pan”) are also frequent in AD [30]. Far more errors occur when the words allow for such errors. Therefore, migratable word pairs, i.e., word pairs in which each letter migration between words (that preserves within-word letter position) creates an existing word, are the most sensitive stimuli to identify AD.

Another observed deficit stemming from a deficit at the early, prelexical stage is the neglect of one side of words (usually their left side). Such a deficit, called Neglect Dyslexia, or “Neglexia”, results in omissions, substitutions, or additions of letters on one side of the target word, which occur more frequently when the resulting error is an existing word. Developmental left Neglect Dyslexia has been reported in Hebrew [31,32,33,34,35] and Arabic [21]. Developmental right Neglect Dyslexia has been identified in Turkish [25]. The most sensitive stimuli are words and nonwords in which letter errors on the neglected side create other existing words. 

The output of the early orthographic–visual analysis is an abstract representation of ordered letter identities. When such representation is inconsistent with the written string, due to an impaired orthographic–visual analysis, reading errors are produced. If such errors cannot be referred to any of the specific mechanisms previously described (letter identification, letter position coding, letter-to-word binding), they are considered “visual errors” (e.g., ARTICHOCK read as “architect”, as in Marshall and Newcombe [36]). According to Morton and Patterson [37], visual errors are defined as substitutions, omissions, and additions of some of the letters of the target word (when at least half of the letters in the error are included in the target word). Reading profiles characterized by a high percentage of visual errors, which are not consistent with one of the specific dyslexias in the orthographic–visual analyzer, are described as Visual Dyslexia (or dyslexia of the output of the orthographic–visual analyzer). Two cases of developmental Visual Dyslexia were reported by McCloskey and Rapp [38] and by Valdois, Gerard, Vanault, and Dugas [39]. A deficit in the orthographic input buffer, an orthographic short-term memory store that holds the orthographic representations until they proceed to the lexical and sublexical routes and parses the string to graphemes and morphemes, is called orthographic input buffer dyslexia. It differs from Visual Dyslexia in that it is also sensitive to length effect and results not only in visual errors, but also in morphological errors. 

The representation activated in the orthographic input buffer is processed through the two routes described by Coltheart et al. [2], i.e., the lexical and sublexical pathways. The lexical route is triggered when the information from the orthographic buffer feeds forward enough activation to the orthographic input lexicon to lead to the recognition of the word representation matching a printed stimulus (e.g., STOMACH). Such representation is linked to the corresponding phonological representation in the phonological output lexicon, which allows a reader to obtain the correct pronunciation of the target word, even in the case of irregular words (e.g., [stomak]). In this case, sight word reading can be applied, so a high reading fluency is observed, in particular when targets are high-frequency words. In this case, word length does not affect reading performance, as whole-word recognition occurs. 

If the written stimulus is a nonword or a new word, never seen before by the reader, or a low-frequency word, the information forwarded from the visual analyzer is not enough to activate any lexical unit, and the only way to read the string of letters aloud is the use of the sublexical route. In this case, the letter string is converted to phoneme strings using grapheme-to-phoneme conversion rules applied at the single letter as well as groups of letters. 

When reading aloud is based only on this sublexical route, errors occur when reading aloud irregular words and decoding is slow, affected by word length. A deficit in the lexical route, i.e., a lack of availability/accessibility of word representations in the orthographic input lexicon or in the phonological output lexicon or a disconnection between these lexicons due to developmental or acquired disorders, is called Surface Dyslexia [40,41,42,43,44,45,46,47,48,49,50,51,52,53,54,55,56,57,58,59,60,61,62,63,64,65,66]. In Surface Dyslexia, the reading of irregular words is impaired, but the decoding of nonwords and the reading of regular words are preserved. Typically, regularization errors are observed in reading words with unpredictable and ambi-phonic graphemes (e.g., the letter “i” in KID [kɪd] and in KIND [kaɪnd]). Moreover, in reading words with three or more syllables, stress assignment may be impaired since in Italian and in some other languages it is lexically determined and not orthographically marked. 

A deficit in the orthographic input lexicon affects not only reading aloud, but also reading comprehension. In the case of regular words, which can be read accurately through the sublexical route, reading aloud would be correct, and the correct representations in the semantic lexicon can be activated by the phonological input. However, it is liable to fail in irregular words, with homophones (KNIGHT/NIGHT), and with so-called potentiophones [48], words whose sublexical reading can result in another existing word (e.g., COME, which may be read to sound like “comb”). If the output of the orthographic–visual analyzer does not activate any lexical representation (input Surface Dyslexia), then it is also not possible to perform a visual lexical decision task, when pseudohomophones (e.g., *ANSER) are administered along with words. In the case of spared orthographic input lexicon (and access to it from print), the lexical decision is spared. 

The sublexical route may also be specifically impaired: this disorder is called Phonological Dyslexia [52,67]. In this case, the reading of known regular and irregular known words is preserved, but when reading nonwords, a slow reading rate and a high rate of errors with several lexicalizations of the letter string and “I do not know” responses are observed [43,68].

From the analysis of the error types produced when reading, it is possible to identify different loci of damage to the sublexical route: Letter-to-Phoneme Conversion Phonological Dyslexia, when there is a failure in sounding out single letters, and Multi-letter Phonological Dyslexia, when errors are specifically associated with the application of multi-letter conversion rules, such as the conversion of digrams (like *CH*, in Italian) and the conversion of letters in specific positions and context-sensitive rules (e.g., in English, the conversion of a vowel preceding a consonant and an *e*, such as in the nonword *NADE). 

More recently, Friedmann and colleagues described other types of deficits that can refer to specific impairments in the sublexical route: Vowel Dyslexia, in which readers omit, substitute, transpose, and add vowel letters in nonwords [69,70,71]. In cases where the person with Vowel Dyslexia also has Surface Dyslexia, they will read not only nonwords but also real words via the sublexical route, and the result would be vowel errors also in real words. Thus, to detect Vowel Dyslexia, nonwords should be presented that, when read with a vowel letter, would create existing words.

Other types of dyslexias, which were already described but require further evidence to be well identified, are the selective deficit of the voicing feature (dyzlegzia), which was already described in writing [72], and a deficit in the nasality feature (nasalexia) [73]. 

Additionally, a deficit in the sublexical route may be a deficit in grapheme-to-phoneme conversion, but it may also be a deficit in the phonological output buffer. The phonological output buffer is the component in which phonemes that are the product of the conversion (and also phonemes arriving from the lexical route) are stored for a short time and assembled into a whole word or a nonword [68,70]. The phonological output buffer is involved in any word and nonword production but is especially sensitive when reading nonwords, which cannot receive lexical feedback from the phonological lexicon in long items, as they may exceed the limit of the number of phonemes it can hold, and in morphologically complex stimuli because it holds morphological affixes and assembles them with the word bases [74].

In the case of Phonological Output Buffer Dyslexia, errors occur not only when reading aloud nonwords but also when reading morphologically complex words and long real words. It also affects word repetition and oral production. Hence, impairment in such a component is expected to also affect word reading, word repetition, picture naming, and speaking. The deficit affects reading only when reading aloud, whereas written word recognition and comprehension are spared.

A different kind of dyslexia, Deep Dyslexia, comes from a combined deficit affecting both the sublexical route and the connection between the lexicons in the lexical route. In this case, words can be read only through the associated meaning stored in the semantic system. High-frequency words with high-imageability features are likely to be read with a higher level of accuracy than abstract words and function words, whereas the reading of nonwords is severely impaired. Such a reading profile was described in adults with acquired deficit [10,75] and also in developmental dyslexia for English [76,77,78] and for Arabic [21]. Evidence of this dyslexia type was also reported in several other studies in English and in Italian contexts [21,36,59,79,80,81,82,83,84,85].

### Aims

The opportunity to link an observed pattern of errors to specific deficits in reading is relevant to the early detection of difficulties that can negatively affect learning to read fluently. Moreover, a detailed assessment of reading mechanisms can provide clues for planning targeted treatments and suggest compensative strategies when the expected improvement in reading skills does not occur. The information drawn from the usual comparison between error rates in reading words and nonwords cannot be enough to discriminate within the different mechanisms described by the extended reading model and the broad range of dyslexias it dictates [1]. 

Thus, in the present study, a new reading battery is described (*Tiltan-IT*) that was specifically developed to provide a detailed assessment of reading skills in Italian school-age children. In particular, such a new measure has two main aims: (a) guiding the clinical observation of reading deficits according to an evidence-based model like the DRC model and assessing reading improvement after specific logotherapy treatment; (b) verifying the cross-linguistic validity of the dual-route model itself. According to the first aim, we provide normative data for Italian children attending primary and middle schools (from second to eighth grade) and the way the various kinds of errors change over time. As for the second aim, we describe the types of dyslexias we identified in Italian using this test.

From an applied perspective, *Tiltan-IT* is expected to fill the gap between the usual behavioral measures of reading ability (speed and accuracy) and the detailed description of reading processes provided by neuropsychological models of reading such as the DRC model. The specific selection of the items and the detailed classification of the errors described in the *Tiltan-IT* offer the opportunity to obtain insights on the functioning of eachmechanism involved in reading aloud, beyond the global level of efficiency of the process in terms of speed and accuracy, usually assessed through text reading tests or word and nonword lists. 

From a research perspective, as the *Tiltan-IT* was developed referring to a model of reading that can be used to describe several different alphabetic languages, it is expected to provide us with the possibility to detect the wide variety of dyslexias described in other languages, like Hebrew, Arabic, English, and Turkish, also in a shallow orthography language like Italian. Such a comparison can be provided, as the different instruments used to assess reading skills in each language were developed according to the same criteria for the item selection and for the error coding used in the original version of the instrument, *Tiltan*, the test battery developed by Friedmann and Gvion [3] for Hebrew.

## 2. Materials and Methods

### 2.1. Participants

In order to collect normative data on the new *Tiltan-IT* battery, 618 Italian-speaking children (2nd–8th graders) were recruited at their schools (Table 1), in the north-western part of Italy. The socio-cultural context they come from can be described as working and middle class. Only 1 child, in 5th grade, had been diagnosed earlier with dyslexia. 

This research was approved by the Ethical Committee of the Department of Psychology at the Catholic University of Milan (n. 48/2022) on October 14th, 2022. Only children whose parents gave their consent for participation in this research (according to the GDPR—EU Reg. 2016/679) were included in this study. 

Beyond the *Tiltan-IT*, participants were presented with measures routinely applied in the Italian context for the diagnosis of Specific Learning Disorder, following the recommendations of the Italian guidelines for the diagnosis of dyslexia [86]. The guidelines recommend the assessment of cognitive functioning—as the definition of Specific Learning Disorders, also reported in the DSM-5 [87], requires that cognitive development is within normative range—and measuring reading ability through the reading of a text and of word and nonword lists. According to these recommendations, Raven’s progressive matrices (*Coloured Progressive Matrices* until 5th grade; *Standard Progressive Matrices* for grades from 6th to 8th) [88,89], *MT Reading Test* [90,91], and Zoccolotti et al.’s *Word and Nonword Reading Test* [92] were administered.

In the *MT Reading Test*, participants are required to read aloud a meaningful passage within a 4 min time limit. Speed (measured as the number of syllables read by seconds) and accuracy (i.e., the number of errors, adjusted for the amount of text read) are the variables used to evaluate reading ability. Passages (and corresponding reference norms) vary depending on school level.

Zoccolotti et al.’s reading test [92] consists of two lists of nonwords (short and long) and four lists of words, corresponding to each combination of two psycholinguistic variables: word frequency (high vs. low) and word length (short vs. long). The measures recorded for each list of stimuli are speed (in seconds) and accuracy (i.e., the number of reading errors). 

Descriptive statistics carried out on the data obtained from the administration of the Raven’s tests and the text reading test revealed that the participants in this study can be considered representative of a normative population (Table 2), as the Raven’s scores are in line with the Italian validation [88,89] and the z-scores referring to reading ability are all around the mean value for each parameter.

### 2.2. Materials: The Tiltan-IT and Its Rationale and Structure

The new Italian dyslexia battery, *Tiltan-IT*, is aimed at providing the opportunity to identify specific impairments, corresponding to the single components of the DRC model and to the different types of dyslexias. The main principles that guided us in the development of this test were (a) the use of stimuli that are sensitive to each type of dyslexia and (b) a detailed error analysis that encompasses the various error types characteristic of each kind of dyslexia. 

The selection of the stimuli was based on the theoretical and empirical knowledge of the various types of dyslexia. When the word frequency was a criterion for item selection, two different corpora were used: the *Italian frequency lexicon of written language* (*CoLFIS*) [93], which is based on a balanced corpus of over 3 million words, reflecting the reading habits of the Italian population, and the *Elementary Lexicon* [94], taken from the frequency count over 1 million occurrences of words in texts read and written by Italian children. 

Here, we describe each dyslexia type we aimed to identify, following the DRC model, and the types of stimuli that are most sensitive to them, which we included in the *Tiltan-IT* (the numbers of items of each type are provided in Table 3). 

To assess the types of impairment that may occur in the various mechanisms of the orthographic-visual analysis, we relied on the following considerations:

(a) Some individuals with Letter Identity Dyslexia [7,11,13] make most errors when the substitution or omission of a letter creates other existing words (e.g., *stalla*, barn → *spalla*, shoulder). In other words, reading errors produce orthographic neighbors. The selection of the stimuli was based on the orthographic neighborhood database [95] developed at the University of Padua. Even in this case, the *CoLFIS* [93] and *Elementary Lexicon* [94] were used to select the less frequent word within a pair of competitors. All the words and nonwords used to detect Attentional Dyslexia, Neglect Dyslexia, and the different types of Phonological Dyslexias can also be instrumental in detecting this dyslexia.
(b) Individuals with Letter Position Dyslexia (LPD) [15,16,17,18,19,20,21,22,23,24,25,26] make most of their errors in migratable words and nonwords, i.e., words and nonwords in which letter transposition within a word creates other words. Therefore, the *Tiltan-IT* included such words and nonwords. The first step of the development of the list of migratable words was the selection of pairs made of a word and its existing-word anagrams (e.g., *corpo,* body, and *copro*, I cover). Because more errors occur when the result of a transposition is of higher frequency than the target word [17], for each pair, the frequency of use of the two words was checked through the corpora reported above, and the less frequent word was included in the *Tiltan-IT* list for detecting LPD.
(c) Individuals with Attentional Dyslexia (AD) [27,28,29,30] make most of the between-word migrations in migratable word pairs, i.e., word pairs where the migration of a letter from one word to another may generate existing words (e.g., goat coal → coat goal). The selection of the items for the word pairs list was made through the orthographic neighborhood database cited above. To increase the sensitivity of the word pairs, we followed the results of Friedmann et al. [28], similarly to the findings of Shallice and McGill [96], Mozer [97], and McClelland and Mozer [98] for normal reading in short exposure, and selected word pairs in which the two words within a pair had the same length and two shared letters. In order to have the highest probability of finding migratable words, only disyllabic, 4–5-letter-long words were considered, as the neighborhood size is the highest for such words within the Italian lexicon. Starting from a word (e.g., *cena*, dinner), we found another word (e.g., *pera*, pear) to obtain a pair where the (within-word position-preserving) migration of the letters between the two words could produce other existing words (e.g., from the migration of the first letters: *cena pera*, dinner pear → *pena cera*, pain wax; from the migration of the third letters: *cena pera*, dinner pear → *cera pena*, wax pain). Stimuli were printed on an A4 paper sheet one pair per line, with a vertical spacing of 8 mm between lines. The *Tiltan-IT* included such migratable word pairs, and it also allowed for vertical migration between words presented in all lists, one above the other.
(d) Individuals with left Neglect Dyslexia [31,32,33,34,35] make errors mainly in words and nonwords in which the omission or substitution of letters on the left side creates another word. In this case, we sorted the lemmas listed in the *CoLFIS* [93] according to their endings, in order to identify sets of words with the same endings (i.e., words that share their right side). From such sets, we selected the longest words, which included other words (e.g., *convento*, convent, which includes *vento*, wind), after checking for the presence of those words in the *Elementary Lexicon* [94].

Beyond the relevant real-word stimuli, selected according to the criteria described above, nonwords are sensitive to all dyslexias of the orthographic–visual analyzer. In particular, LPD was assessed using migratable nonwords, i.e., nonwords that differ from existing words in the position of two letters. Neglexia was assessed using nonwords that differ from existing words on their left side.

To assess the types of impairment that may occur in the lexical route, we relied on the following considerations:

(e) Individuals with Surface Dyslexia [36,37,38,39,40,41,42,43,44,45,46,47,48,49,50,51,52,53,54,55,56,57,58,59,60,61,62,63,64,65,66] make errors mainly in irregular or unpredictable words, i.e., in those words whose reading via the sublexical route is ambiguous or different from the target pronunciation (e.g., island, door, stomach, bear). Due to the regularity of the Italian orthography, ambiguity in conversion is almost exclusively expressed in the stress position of words with more than 2 syllables, which can be either on the penultimate or on the antepenultimate syllable and is not orthographically marked. We thus prepared a list of trisyllabic words with either a penultimate or antepenultimate diacritically unmarked stress position that were balanced for the orthosyllabic structure of the words (e.g., [ˈ*sabato*], Saturday, and [*pa*ˈ*lato*], palate; [ˈ*popolo*], people, and [*pin*ˈ*olo*], pine nut). Stress position errors without any further orthographic or phonological errors were counted.

To assess the types of impairment that may occur in the sublexical route, we used the following principles:

(f) Individuals with Vowel Dyslexia [69,70,71] make errors mainly in nonwords in which vowel letter errors (substitutions, omissions, additions, transpositions) create existing words (e.g., *bredo → *brodo*, broth; *ponna → *panno*, cloth). If readers also have Surface Dyslexia, they make such errors not only on nonwords but also on words, and, therefore, we also made sure that the word list includes such target words that have vowel-error neighbors along the same lines of selection. Thus, we selected both nonwords and words in which vowel errors create existing words. In the selection of stimuli for this dyslexia too, the selection of the stimuli started from the generation of lists of words paired with their orthographic neighbors and their anagrams, selected through the online databases cited above. The pairs of words differing from each other by a vowel substitution or transposition were chosen. The less frequent word in the pair was selected and included in the word list for the detection of Vowel Dyslexia (e.g., *abate*, abbot → *abete*, fir). The nonword list was created by changing one vowel in the items in the word list (e.g., *abate* → *abote).
(g) Phonological Dyslexias [10,67,68,72,73,74] affecting the consonant conversion (multi-letter, voicing, and nasality) are all manifested in nonwords. They can also be detected in existing words for children who read existing words sublexically (as is the case with Surface Dyslexia). Therefore, *Tiltan-IT* includes a list of nonwords, created by changing one or two letters in real words, in which a letter substitution or migration creates existing words, and words in which a vowel, voicing, or nasality error creates other existing words (to detect Vowel, Voicing, or Nasality Dyslexia, respectively). The test also included words in which failure to apply multi-letter level conversion rules creates incorrect reading.

To assess Deep Dyslexia, we relied on the following types of errors:

(h) Individuals with Deep Dyslexia [21,36,59,75,76,77,78,79,80,81,82,83,84,85,99] make errors mainly in morphologically complex words, in function words, in abstract words, and in nonwords. Additionally, they are likely to produce synonyms of (or semantically related words to) the target stimuli. Correspondingly, in the *Tiltan-IT*, morphologically complex words (e.g., *premettere*, to preface), function words (e.g., *eppure*, nevertheless; *sebbene*, although; etc.), high-frequency abstract words (e.g., *storia*, history), and nonwords were presented. Moreover, to prompt semantic errors, low-frequency words with high-frequency semantically related words were also selected (e.g., *sabbia*, sand → *spiaggia*, beach), as the expected errors were substitutions of the target items with the high-frequency competitors. The selection of the stimuli was made by sorting the lemmas listed in the *CoLFIS*, according to different criteria (grammatical category, word frequency), and by looking for synonyms in the Italian dictionary. Beyond the various dyslexias of the sublexical route, the nonwords are also sensitive for the detection of Deep Dyslexia, in which the sublexical route is impaired.

To assess an impairment in the phonological output buffer we used following guidelines:

(i) Individuals with phonological output buffer impairment [41,42,43,74] have difficulty in producing the correct sequence of phonemes corresponding to the target letter string, so the doubling, omission, migration, and substitution of phonemes are observed. They also make morphological errors and are affected by length and by morphological complexity. Every item of the *Tiltan-IT* can be useful to elicit phoneme errors, and stimuli that are especially sensitive to this impairment are nonwords and long and morphologically complex stimuli. Phonemes that appear twice in the word seem to place a special challenge to individuals with this impairment, so letter doubling errors and omissions of an instance of a doubled phoneme/letter can offer a specific clue on the presence of this impairment.

It is worth noting that all stimuli allow for the detection of Visual Dyslexia and Letter Identity Dyslexia, whereas long and morphologically complex words, which were selected within each of the word lists described above, may detect input and output buffer impairments (as well as deep dyslexia).

A crucial property of the way the *Tiltan-IT* was constructed was that we selected stimuli along the above lines, which could serve for detecting more than one dyslexia type, depending on the error the participant made. For example, the word *from* is a function word, so a substitution with another function word like “of” would indicate Deep Dyslexia or a deficit in the orthographic input or phonological output buffers. But it is also a migratable word, so an error in which the reader says “form” instead could indicate Letter Position Dyslexia or Vowel Dyslexia; substitution with “frog” would indicate Letter Identity or Visual/Buffer Dyslexias, and if the letter ‘g’ appears as a final letter in the neighboring words (e.g., from blog → frog blog), it would indicate Attentional Dyslexia. 

The stimuli were presented in 4 lists, described in Table 3: the (1) word list (including migratable, Neglect, Vowel, and Deep Dyslexia stimuli); (2) stress list (Surface Dyslexia); (3) word pairs list (Attentional Dyslexia); and (4) nonword list (Phonological Dyslexias). The stimuli within each list were printed one above the other on an A4 paper sheet. Items within each list were randomized once, and then, the order was identical for all participants. The order of presentation of the lists was randomized at every administration. Stimuli were presented in lower case, in Arial font 18. 

Reading errors were coded through a coding scheme that classified the errors according to the following aspects: (a) the type of error: substitution, addition, omission, and within-word migration; (b) the type of unit affected: vowel letter, consonant letter, phonological feature (voicing, nasality, sibilants), morphological affixes, and digraphs; (c) the position within the word in which the error occurred (exterior-left, middle, right-exterior); (d) whether or not the error could have emerged from a neighboring word; (e) whether errors derive from sublexical rather than lexical reading of words (stress errors). Each error type was then associated to a specific type of dyslexia, referring to different mechanisms and components involved in reading, as described above.

### 2.3. Procedure

Children were assessed by undergraduate students, native speakers of Italian, who undertook specific training for the administration of the tests. Tests were administered individually in a quiet room at school in two sessions of about one hour—one for the Raven’s progressive matrices [88,89] and the *Tiltan-IT* battery and the other for the *MT Reading Test* [90,91] and for Zoccolotti et al.’s reading test [92]. Readings were recorded, and measures of speed and accuracy were obtained for the *MT Reading Test* and for Zoccolotti et al.’s *Word and Nonword Reading Test*. Errors that occurred in the reading of the *Tiltan-IT* battery were transcribed verbatim and coded by two raters (C.L. and D.T.); in case of disagreement, a third rater (N.F.) was engaged as a referee. All responses were coded and analysed, including errors that were later corrected. 

### 2.4. Analyses

The first aim of this study was to develop normative data for the new Italian dyslexia battery, designed to detect the different types of dyslexia described by Friedmann and Coltheart [1]. To reach this aim, the following analyses were carried out:

(a) Errors produced in reading the *Tiltan-IT* lists were coded, and the frequency of each code was computed for each child. When the error could be described in two different ways, it was coded in “or” coding. Each error could be coded with up to three different alternative codes (e.g., *prati*, lawns → *pirati*, pirates could be a vowel addition error or it could be a vowel migration from the word “*circo”* (circus) below, indicating Attentional Dyslexia); in case of several alternative codes, the score could be 0.5 for each alternative when there were two options or 0.33 for each alternative when three codes were assigned to the same error.
(b) Other reading errors could involve several errors in the same word, e.g., *vondesta was erroneously read as *voldestra with two errors, i.e., consonant substitution and consonant addition. When several errors were detected in the same word, they were coded in “and” coding.
(c) Clusters corresponding to different types of dyslexias were obtained by summing up the number of the codes related to each type of dyslexia, e.g., in the Vowel Dyslexia cluster, the following error types are summed together: vowel substitution (e.g., *abate*, abbot → *abete*, fir), vowel addition (e.g., *carta*, paper → *carota*, carrot), vowel omission (e.g., *solido*, solid →*soldo*, money), and all other errors in which vowels are involved (migration within and between words, vowel doubling). The following error clusters were obtained: Attentional Dyslexia (AD), Letter Position Dyslexia (LPD), Surface Dyslexia, Multi-letter Phonological Dyslexia (errors in the sublexical conversion of digraphs or other rules applying to more than one letter), Vowel Dyslexia, Consonant Dyslexia (consonant substitution, omission, addition, nasalization, cluster omission), Voicing Dyslexia, Doubling of a letter in the word or omission of an instance of a letter that appears twice in the word (which may be related to Phonological Output Buffer Dyslexia), and Deep Dyslexia.
(d) After removing outliers, the mean, *SD*, and threshold according to Crawford and Howell’s [100] *t*-test, with a two-tailed probability of 0.005, were computed for each cluster to provide normative data for each grade (2nd to 8th) of the Italian population. When the computed threshold was smaller than 2 errors, in cases where the controls made no errors of this kind or very few of them, a minimum threshold of 2 errors was used.
(e) When the trend of normative data showed unexpected values, *c-NORM*, *Continuous Norming* [R package] [101], was applied through the *jamovi* (2.3.28) software [102].

The second aim of this study was to verify the possibility of identifying different types of dyslexias also in a shallow orthography language like Italian. To this aim, the performance of each participant was compared to the normative data in each of the error clusters, with an alpha of 0.005, and the type of dyslexia was determined accordingly. This allowed us to collect the frequency of the different types of dyslexias in our sample. 

## 3. Results

### 3.1. Normative Data 

All cluster scores (AD, LPD, Surface Dyslexia, Multi-letter Phonological Dyslexia, Vowel Dyslexia, Voicing Dyslexia, Consonant Dyslexia, Doubling errors) were summed up for words (word list and stress list together), word pairs, and nonwords separately, and the overall error score was computed. The mean, SD, and fifth percentiles of the distribution of each index were computed for each grade. The data of children whose error scores were higher than—or equal to—the fifth percentile in at least one of the four indexes (words, word pairs, nonwords, overall) were removed from the database. Following this criterion, 57 children (9.2%) were considered outliers, and norms were established on the data from the remaining 561 children (Table 4).

**Figure 2:**
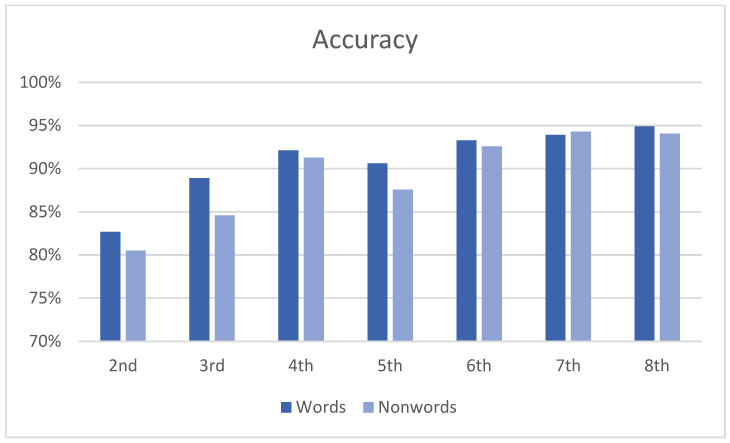
Percentages of accuracy for reading aloud words and nonwords by grade (N = 561).

**Figure 3:**
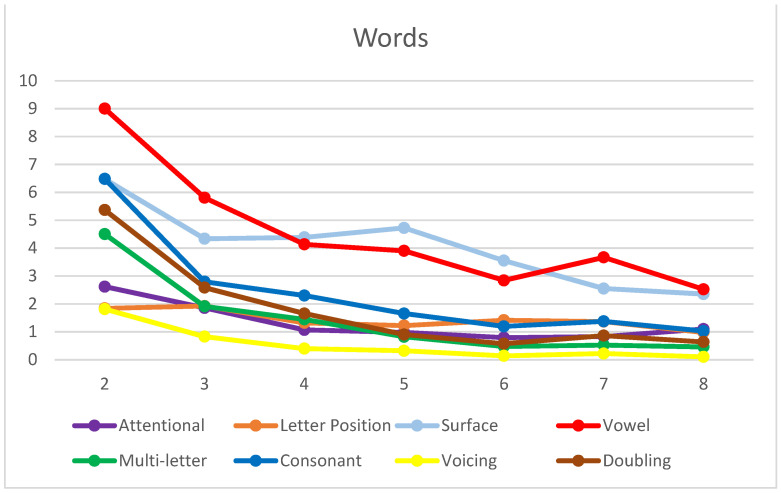
Words: mean error scores in each cluster by grade (N = 561).

**Figure 4:**
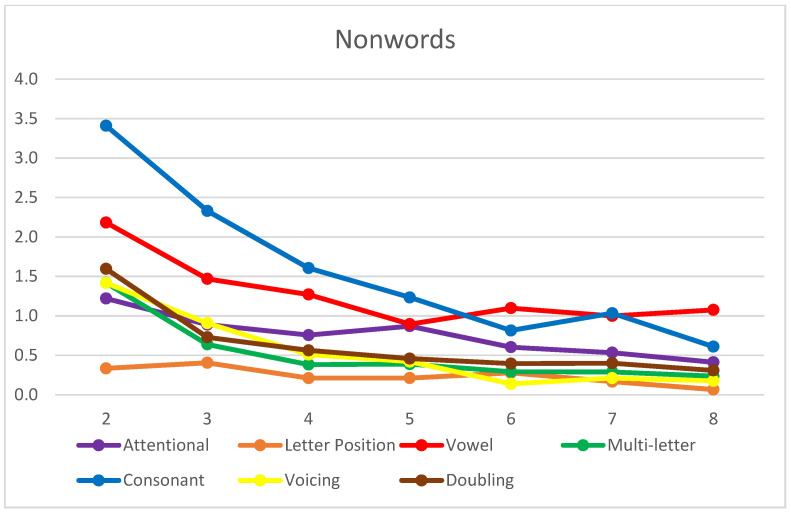
Nonwords: mean error scores in each cluster by grade.

Overall, a high level of accuracy was observed in reading both word (91%) and nonword (89%) lists, with an increasing trend from second to eighth grade, as expected (Figure 2). When considering the clusters of errors coded by the Tiltan-IT coding scheme, a similar picture could be observed for both words (Figure 3) and nonwords (Figure 4) in terms of the overall improvement in reading.

In both the words and nonwords charts (Figure 3 and Figure 4), it is possible to detect a clear decreasing trend in error scores from second to fourth grade. However, it is worth noting that decoding—i.e., the application of GPC rules (vowel, multi-letter, consonant, and voicing) to read aloud—seems to be acquired early in Italian children (Figure 4), as from the third grade, all the types of errors are below a mean of two occurrences. The number of phonological errors in consonant and vowel conversions sensibly decreases from third to fourth grade. In contrast, word recognition (assessed through the stress list) is likely to be mastered only in middle school, as the trend in stress errors seems to suggest (Figure 3). 

Descriptive statistics of error scores, coded in each cluster, were carried out by grade, and the application of Continuous Norming (c-NORM: https://CRAN.R-project.org/package=cNORM, accessed on 15 January 2024) [R package, version 4.1] [101], through the jamovi software [version 2.3] [102], allowed us to provide cutoff values corresponding to the expected trend in school children’s reading ability. Table 5 and Table 6 summarize the error rates in each error cluster, in each grade, for the words and nonwords, respectively. They also provide the Crawford and Howell’s *t*-test [100] cutoff, identifying the error rate of a participant that is significantly higher than the norm for their age (at 0.005 *p*-value) (the choice for such a high-level *p*-value comes from the need to reduce the probability of false positives due to the high number of comparisons made to describe the reading profile of each participant).

Table A1 and Table A2 in Appendix A provide further information on the fifth and tenth percentiles in each grade, and Table A3 provides this information for the combined error rate in the word and nonword lists. 

The presence of error scores higher than such cutoff values in some clusters suggests probable, specific difficulties in one or in several reading mechanisms. The last two columns of Table 5 refer to the stress list and to the word pairs list, which were specifically devised to identify Surface and Attentional Dyslexia, respectively. The values of such columns provide more definitive evidence of the corresponding types of dyslexias, beyond the values provided by the first and the third clusters of the same table. Neglect Dyslexia and Deep Dyslexia clusters were not reported within the normative tables, as the error scores were not higher than two in any of the age groups. 

### 3.2. Identification of Different Types of Dyslexias

**Figure 5:**
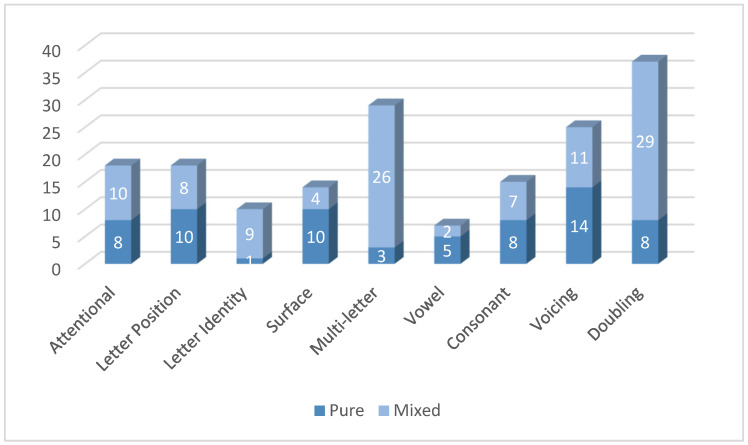
Number of participants whose number of errors in the specific error clusters is higher than Crawford and Howell’s [100] thresholds (*p* < 0.005). The darker shade of blue in the bottom part of each column represents the number of pure cases of this dyslexia, and the lighter, top part of the column indicates the number of participants who had this dyslexia in addition to at least another type of dyslexia.

The next step of the analysis was the identification, in the original database (N = 618), of performance scores that were significantly poorer (corrected for multiple comparisons, *p* < 0.005) than those of the normative group. All error scores higher than Crawford and Howell’s [100] threshold were marked, and the number of children who were characterized by each error cluster, either in a pure or in a mixed type of dyslexia, was computed (Figure 5) (for example, if a participant had pure attentional dyslexia, she is presented in the bottom part of the attentional dyslexia column. If she also had surface dyslexia errors, she appears both in the top part of the attentional dyslexia column and in the top part of the surface dyslexia column).

**Figure 6:**
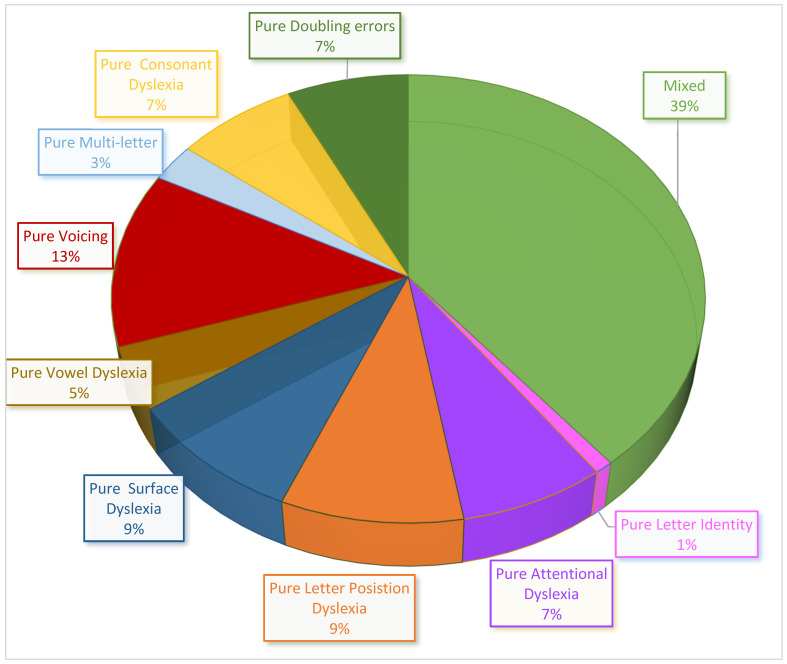
Percentage of pure types of dyslexia (61%) that affect only one component in the reading process and percentage of children who show poor performance in more than one cluster of reading errors (39%), from all the children who performed significantly below norms (*p* < 0.005) in at least one error cluster (N = 110).

The *Tiltan-IT* was designed to include several sets of stimuli that would be most sensitive for the detection of different types of dyslexias. Figure 5 shows that using these stimuli, the aim was achieved, and various dyslexia types were represented. For nonwords, no stress errors (related to Surface Dyslexia) are reported, as penultimate and antepenultimate stress assignments (e.g., [*sar’cone] or [*’sarcone]) would both be plausible in reading nonwords. As for deficits in the sublexical route, such as Vowel Dyslexia, deficits in converting consonants, and doubling (which may indicate Phonological Output Buffer Dyslexia), they are more represented in the nonword list than in the word list. The number of children who showed several types of dyslexias (mixed profiles) and the number of children who have a pure type of dyslexia are reported in Figure 6.

#### Types of Dyslexia That Were Identified in the Group

Out of the 618 participants tested, 110 (18%) obtained an error score higher than Crawford and Howell’s [100] cutoff in at least one cluster. Sixty-seven children (10.8% of all 618 tested children; 61% of the participants with reading performance below norms) showed a specific impairment in only one error cluster.

Of the participants who showed a pure kind of dyslexia, we identified: 

10 children with Letter Position Dyslexia, 

8 with Attentional Dyslexia, 

1 with Letter Identity Dyslexia, i.e., a specific impairment in reading consonants both in words and in nonwords, 

10 children with Surface Dyslexia, 

14 children with Voicing Dyslexia, 

5 with had Vowel Dyslexia

3 with Multi-letter phonological Dyslexia, 

8 with Consonant Dyslexia, 

8 who made an atypical number of doubling errors. 

The other 43 children (6.9% of all children tested) showed an overall performance that was significantly below the normative mean in several error clusters (Figure 6). 

Notice that the children who made significantly more errors in consonants than the norms could have a deficit also in vowels or a deficit that is not necessarily based on the sublexical route—the impairment could also result from a deficit in the orthographic–visual analysis, in the orthographic input buffer, or in the phonological output buffer. A further analysis of the participants who showed a consonant deficit indicated a subgroup of three children who probably are selectively impaired in the sublexical route: they showed a very selective pattern of deficit in consonant letters only in nonwords, but not in words, and only in consonants, but not in vowels. Five other children showed the complementary deficit in the sublexical route: a deficit in reading vowel letters only in nonwords and only in vowels but not in consonants.

### 3.3. Comparison with the MT Reading Test [90,91] and with Zoccolotti et al.’s Word and Nonword Reading Test [92]

In order to verify whether the *Tiltan-IT* is in line with the sensitivity of the other tests usually applied for the assessment of reading disorders, the results obtained with the new battery were compared with the data from the administration of the *MT Reading Test* [90,91] and the *Word and Nonword Reading Test* by Zoccolotti et al. [92]. In Italy, the latter test is often used in diagnostic protocols to assess reading ability, after the administration of text reading, as it was developed to identify specific difficulties in decoding and in lexical access. The cutoff value used to identify children with dyslexia (CwD) in both tests was the fifth percentile for each grade, according to the cutoff values suggested by the *International Statistical Classification of Diseases and Related Health Problems* (ICD-10) [103] for the diagnosis of Specific Reading Disorders. 

Zoccolotti et al.’s test [92] identified 122 children (20%) whose performance was significantly below the norms in at least two lists in the battery. Such data are in line with the results emerging from the *Tiltan-IT*, as this test also provided a similar rate of reading disturbances (17.8%) (χ^2^ = 57.71; *p* < 0.001) (Table 7).

This suggests that the *Tiltan-IT* is as sensitive as Zoccolotti et al.’s test, and beyond the identification of whether a child has dyslexia or not, the *Tiltan-IT* provides information about the exact type of dyslexia each participant has. 

As for the comparison with the text reading test, the *MT Reading Test* [90,91] identified only fifteen children out of six hundred and eighteen (2.4%) with error scores higher than the cutoff and only eight children (1.3%) were impaired in reading accuracy also in the *Tiltan-IT* battery, suggesting a diagnosis of dyslexia (χ^2^ = 13.26; *p* < 0.001) (Table 8). This large difference between the rate of dyslexia identified with word and nonword list tests (Zoccolotti et al.’s test, *Tiltan-IT*) and the text reading test indicates the far greater sensitivity of the word/nonword list tests.

## 4. Discussion

### 4.1. Evidence for Multiple Types of Dyslexias Identified with the Tiltan-IT Test

This study relied on the detailed neuropsychological framework for the reading process described in [1] to develop a reading test that would be able to identify the various types of developmental dyslexias also in Italian (*Tiltan-IT*). The main rationale was that if there are various components in the reading process, each of them can be impaired, giving rise to a specific type of dyslexia. Given that each type of dyslexia has its own properties with respect to stimuli (words and nonwords) that are most sensitive to each dyslexia type and with respect to error types, we developed a test that includes stimuli sensitive to each kind of dyslexia and applied a detailed analysis of the types of errors that each participant produced. 

The data from the administration of the *Tiltan-IT* to a sample of 618 children attending primary and middle school allowed us to observe the production of the expected pattern of errors. Descriptive statistics of the error scores were carried out for each error cluster, to obtain normative data. Such data were useful to set cutoff values, discriminating levels of performance significantly poorer than the typical ability for each grade (from second to eighth grade). After marking all poor performance scores, it was possible to identify children who showed a significantly higher occurrence of errors in a specific error cluster. Such specific deficits were interpreted as evidence of the possibility to detect specific impairments in single mechanisms of the reading process that can refer to different types of dyslexias. In more detail, the performance of 67 (10.8%) children involved error clusters that are specific for one type of dyslexia. When counting only participants who showed a selective deficit yielding a single pure type of dyslexia, ten showed pure Letter Position Dyslexia (18 had Letter Position Dyslexia in addition to another dyslexia), eight Attentional Dyslexia (18 had Attentional Dyslexia in addition to another dyslexia), one Letter Identity Dyslexia, ten Surface Dyslexia, five Vowel Dyslexia, and in 33 children dyslexias involving consonant conversion were also observed, including fourteen Voicing Dyslexias, three Multi-letter Dyslexias, eight Consonant Dyslexias, and, in eight cases, an atypical number of Doubling errors. Additional 43 children (6.9%) showed a mixed pattern of several reading impairments. Whereas Surface Dyslexia has already been consistently reported for Italian [66], the other dyslexia types form the first report of these dyslexia types in Italian. This is the first report of Letter Position Dyslexia, Attentional Dyslexia, Vowel Dyslexia, Voicing Dyslexia, and Multi-letter phonological Dyslexia in Italian. 

Using this approach, we were able to identify in Italian, a shallow orthography language, the various types of developmental dyslexias that have already been described in several languages. This evidence supports the cross-linguistic validity of the DRC model and the error classification developed by Friedmann and Gvion [3] for Hebrew.

Furthermore, this report of a selective developmental deficit in reading consonants in nonwords is the first in the world. Until now, there have been reports of a selective deficit in the sublexical processing of vowel letters [69,70,71]. Here, we found three participants who had a selective deficit in the conversion of consonant letters: they made consonant errors significantly more than the control threshold but not vowel errors, in nonwords but not in words. This finding, which should be further studied, supports the idea that vowel letters and consonant letters are converted separately in the sublexical route.

### 4.2. Application of the Tiltan-IT in the Reading Assessment

The data provided by the administration of the *Tiltan-IT* and by the scoring of the reading errors were compared with the results of two other reading tests often applied in Italy, the *MT* text reading test [90,91] and the *Word and Nonword Reading Test* by Zoccolotti et al. [92].

The results of the comparison with the text reading test suggest that the contextual meaning of the text passage can offer an important cue for decoding words, so text reading is a useful tool to assess reading ability in an ecological context. However, it is not sensitive enough to detect specific impairments in the reading process, and such a limitation must be taken into consideration. In fact, even though the presence of a specific impairment can be compensated by different strategies used by the child during primary school, it can significantly affect the achievement of the individuals when they face complex texts and have a large number of pages to read when taking exams in college and high school. For these reasons, the Italian guidelines recommend the use of lists of words and nonwords. 

Zoccolotti et al.’s test [92] allows clinicians to evaluate both the use of the sublexical route, through the observation of the performance in reading nonword lists, and the lexical route, which can be inferred from the fluency in reading high- and low-frequency words. Hence, from the administration of the *Word and Nonword Reading Test*, Surface Dyslexia and Phonological Dyslexia can be detected. 

The percentage of children with dyslexia (CwD) identified by the *Tiltan-IT* is in line with the percentage of CwD identified by the *Word and Nonword Reading Test*, but the new *Tiltan-IT* battery offers the opportunity to describe specific types of impairment not only in the sublexical route (Multi-letter Phonological, Vowel, and Consonant Dyslexias, voicing, doubling errors) and in the lexical route (Surface Dyslexia), through the rate of stress assignment errors, but also in the early stages of visual stimulus processing (Letter Identity Dyslexia, Letter Position Dyslexia, and Attentional Dyslexia). Moreover, in the *Tiltan-IT* battery, there are items aimed at detecting Neglect Dyslexia and Deep Dyslexia, which are quite rare in developmental dyslexia. This consideration suggests that the administration of the *Tiltan-IT* battery can be useful in providing a detailed picture of the reading ability of the children, as it offers a lot of information beyond the description found through the *Word and Nonword Reading Test*. In other words, the new test provides the clinicians the opportunity to identify specific impairments underlying poor reading performance, which might not be detected by the usual tests applied to make the diagnosis of developmental dyslexia, and provides useful information for the treatment of reading difficulties and disturbances.

### 4.3. Implications of Findings Regarding Types of Dyslexias for the Debate on the Mechanisms Underlying Reading Performance

The vast majority of studies that attempted the interpretation of the impairments underlying reading difficulties have recurred to unitary explanations of dyslexia by suggesting a “core deficit”, which would offer a single explanation common to every case of reading impairment. Several explanations were proposed. According to the *phonological hypothesis*, children with dyslexia have difficulty with phonetic and/or phonological discrimination [104], whereas the *temporal perception hypothesis* suggests that a poor discrimination between short-duration stimuli in rapid succession is the reason for reading deficits and difficulties in nonword repetition tasks, which can be observed in children with dyslexia [105,106]. More recently, a *visual hypothesis* was proposed, according to which the “core deficit” of dyslexia is a processing impairment along the magnocellular visual pathway (the so-called “where/how” pathway) [107,108], which enables the processing of the spatial location of visual stimuli and, particularly, the processing of transient stimuli: impaired development of the magnocellular system would cause the instability of visual images in reading [107,108]. In readers with dyslexia, this instability of visual images would cause confusion in the letter order, resulting in a processing deficit in the visual form of words and an impediment in the acquisition of spelling skills. Also, the *spatial attention disorder* hypothesis suggested by Facoetti et al. [109,110] is focused on the magnocellular pathway: according to such a theory, readers with dyslexia would suffer from a reduced ability to direct attention voluntarily or automatically to spatial visual stimuli (e.g., Posner’s paradigm). Another visual hypothesis suggests that developmental reading disorders depend on the *crowding effect* [111,112,113], as in readers with dyslexia there would be a disproportionate enlargement of the minimum distance required to separately perceive neighboring stimuli. The result would be a deficit in word shape identification and the abnormal acquisition of spelling knowledge. A different explanation of dyslexia comes from the *cerebellar theory*, according to which the core deficit of dyslexia would be in automatization processes. In fact, the cerebellum plays an important role not only in motor control and, in particular, in speech articulation but also in the automatization of rapidly sequenced motor skills, such as keyboard typing and reading aloud [114,115]. 

Similar unitary explanations of dyslexia come from functional neuroimaging studies that have proposed a single (or predominant) processing area [116] and/or a predominant dysfunctional area in developmental dyslexia [117,118]. However, systematic reviews on the brain regions associated with developmental dyslexia [119,120] provided evidence that the reading process involves several cortical networks, which can be selectively impaired. In the case of developmental disorders, a low level of specialization of such networks was observed, along with a low level of activation of the areas typically devoted to letter string perception and recognition. 

The current results, indicating different kinds of dyslexia, each stemming from a deficit in a different stage of the reading process, suggest that a unitary explanation for all kinds of dyslexia is impossible and that studies of the underlying deficit in dyslexia, as well as the neural underpinnings of dyslexia, should instead target dyslexias, in plural, and the different underpinnings of the different types of dyslexias. 

### 4.4. Limitations of the Present Study and Future Development of the Reading Assessment through the Tiltan-IT

In the current study, we relied on a test of reading aloud, which turned out to be sensitive for the identification of multiple specific types of dyslexias. However, in order to further improve the granularity of identification of the impaired mechanisms involved in each of the various dyslexias, further tests should be used that assess the functioning of each component of the reading process. To evaluate the quality of string representation in the orthographic input buffer, input tasks that do not involve reading aloud (e.g., same–different decision tasks on written stimuli, lexical decision tasks, word-to-picture matching, a semantic decision task) can be administered; other tasks can assess phonological output without reading input to examine an impairment in the phonological output buffer using, e.g., a nonword repetition task, as well as a picture-naming task. Such tasks would allow for making further distinctions in the description of reading performance. For example, for a child who makes surface errors in reading aloud, it is relevant to examine whether his/her deficit is in the orthographic input lexicon or in the orthographic output lexicon. In a similar way, further assessment also allows for the identification of the source of Phonological Dyslexia, as it can suggest that poor reading performance arises either from a deficit in the grapheme-to-phoneme conversion system or from a deficit in one of the buffers. Case studies and the in-depth analysis of selective impairments will allow us to validate the correspondence between error clusters and types of dyslexias we have assumed as an explanation of our results.

## 5. Conclusions

The development and the application of the *Tiltan-IT* reading battery offered the opportunity to detect specific reading impairments that the usual tools administered for the diagnosis of dyslexia cannot identify. So, the new battery is expected to provide relevant information for the diagnosis and the treatment of different types of dyslexias. However, the assessment method, based on the dual-route reading model, must be enriched by further tests aimed at finding evidence on the specific mechanisms involved in reading impairment to become a real and useful support for speech therapists and neuropsychologists. 

From the results reported in this study, the *Tiltan-IT* has great potential in research on the cross-linguistic study of reading processes. The coding system used for the classification of reading errors in Italian was developed according to the same rationale used for similar coding schemes in other languages (Hebrew, Arabic, English, French, and Turkish) that are different from Italian in several aspects. The opportunity to compare the results from several linguistic systems is a great advantage in testing models of reading and obtaining new evidence on the development of reading ability from a cross-linguistic perspective.

## Figures and Tables

**Table 1:** Distribution of participants by gender (M, F) and by grade (2nd through 8th).

Grade	Gender		Total
	M	F	
2	46	36	82
3	26	44	70
4	52	50	102
5	46	42	88
6	73	70	143
7	26	38	64
8	30	39	69
Total	299	319	618

**Table 2:** Nonverbal reasoning and ability in the MT text reading ^a^ [90,91]: descriptive statistics.

		Raven’s CPM-SPM		Speed (Sill/Sec)		Speed (*z* Score)		Accuracy (Raw Score)		Accuracy (*z* Score)	
Grade	N	M	SD	M	SD	M	SD	M	SD	M	SD
2	82	23.8	4.5	1.5	0.7	−0.14	0.50	11.4	8.0	0.25	1.01
3	70	26.3	3.8	2.6	0.8	−0.14	0.67	5.7	4.4	0.16	0.86
4	102	29.6	4.0	3.5	1.0	−0.30	0.19	4.0	3.6	−0.20	0.70
5	88	30.7	4.1	3.4	0.9	−0.19	0.41	6.8	4.9	0.14	0.78
6	143	38.5	8.1	3.9	0.9	−0.03	1.18	7.3	5.6	−0.10	0.78
7	64	40.3	6.3	4.3	0.8	−0.09	0.59	4.9	3.7	−0.31	0.66
8	69	40.7	8.2	5.1	0.8	0.25	0.84	3.4	2.4	−0.12	0.65
Total	618	33.0	8.6	3.5	1.3	−0.10	0.76	6.3	5.6	−0.03	0.81

^a^ The text reading test [90,91] asks for different difficulty and length passages according to grade.

**Table 3:** Types of stimuli in the *Tiltan-IT* and the types of dyslexias they aim to detect °, the number of stimuli of each kind in the list, their mean frequency (for nonwords—their mean N-size), mean number of letters, and an example for the stimulus and a relevant error type.

Stimulus Type and the Dyslexia It Should Detect	N	Frequency	Length	Examples of Items and Expected Errors
*WORD LIST*				
Migratable words—words in which transpositions may create other words (Letter Position Dyslexia, LPD)	20	38	6.25	lardo → *ladro*
Words in which errors on the left side may create other words (Neglect Dyslexia)	40	15	8	incidente → *dente*
Words in which vowel errors may create other words (Vowel Dyslexia)	20	35	5	pazza → *pizza*
Function words and high-frequency abstract nouns (Deep Dyslexia)	30	764	5.93	circa → *quasi*
Words with high-frequency synonyms (Deep Dyslexia)	15	78	5.4	selva → *bosco*
*STRESS LIST* Irregular words with unpredictable stress position (Surface Dyslexia) ^	40	97	6.36	ˈsabato → *saˈbato solˈdato →*ˈ*soldato*
*WORD PAIR LIST* Word pairs in which between-word migration creates existing words (Attentional Dyslexia)	50 (25 pairs)	67	4.48	viso vano → *vino vaso*
*NONWORD LIST*	N	N-size	Length	Examples of items
Pseudowords (Phonological Dyslexias, Deep Dyslexia, Vowel Dyslexia, LPD, and other orthographic analysis dyslexias)	40	5.8	5.6	*rezzo
*Total*	*255*			

° All words allowed for Visual Dyslexia/Letter Identity Dyslexia; long and morphologically complex words within each list may count for input and output buffer impairments. Each item could serve for detecting more than one dyslexia type, depending on the error the participant made. ^ Stress is placed in the syllable after the position marker.

**Table 4:** Distribution of the normative sample by gender (M, F) and grade (2nd through 8th) after the exclusion of outliers.

Grade	Gender		Total
	M	F	
2	41	33	74
3	23	42	65
4	46	47	93
5	41	39	80
6	66	63	129
7	22	34	56
8	27	37	64
**Total**	**266**	**295**	**561**

**Table 5:** Word list: mean scores (and *SD*) for errors identifying each dyslexia type and cutoff values (thresholds according to Crawford and Howell [100], *p* = 0.005) by grade.

Grade	Number of Participants	M (SD) Threshold	Attentional	Letter Position	Surface	Multi-letter	Vowel	Consonant	Doubling	Total Errors	Surface Errors in Stress List	Between-Word Migrations in Word Pairs
2	74	M (SD)	5.4 (3.6)	3.5 (2.5)	6.5 (3.0)	4.5 (4.5)	8.0 (5.9)	6.5 (5.0)	7.9 (6.5)	42.7 (21.5)	4.8 (2.4)	2.6 (2.2)
		threshold	15	10	14	16	23	19	24	96	11	8
3	65	M (SD)	3.8 (3.0)	2.9 (2.1)	4.3 (2.9)	1.9 (2.9)	5.0 (4.9)	2.8 (3.5)	4.3 (4.5)	25.4 (16.6)	3.1 (2.1)	2.0 (2.1)
		threshold	12	8	12	9	17	12	16	66	9	8
4	93	M (SD)	2.4 (2.2)	2.1 (1.8)	4.2 (2.8)	1.4 (2.0)	3.3 (3.3)	2.2 (2.2)	2.5 (2.7)	18.2 (13.0)	3.2 (2.2)	1.3 (1.5)
		threshold	8	7	11	7	12	8	9	50	9	5
5	80	M (SD)	2.3 (1.9)	1.8 (1.7)	4.7 (2.3)	0.8 (1.4)	3.3 (2.7)	1.7	2.0 (1.9)	16.8 (9.9)	3.5 (1.8)	0.8 (1.0)
		threshold	7	6	11	5	10	7	7	41	8	4
6	129	M (SD)	1.6 (1.2)	2.2 (1.8)	3.5 (2.1)	0.5 (0.8)	2.2 (2.0)	1.2 (1.4)	1.3 (1.6)	12.5 (7.0)	2.8 (1.7)	0.7 (0.8)
		threshold	5	7	9	3	8	5	6	30	7	3
7	56	M (SD)	2.0 (1.6)	2.1 (2.0)	2.6 (1.7)	0.5 (0.9)	3.1 (2.1)	1.4 (1.8)	1.9 (1.5)	13.7 (8.3)	2.0 (1.4)	0.6 (0.8)
		threshold	6	7	7	3	9	6	6	34	6	3
8	64	M (SD)	1.9 (1.5)	1.3 (1.2)	2.3 (1.9)	0.4 (0.9)	2.1 (2.0)	1.0 (1.2)	1.2 (1.4)	10.5 (6.5)	1.8 (1.7)	0.6 (0.7)
		threshold	6	5	7	3	7	4	5	27	6	3

**Table 6:** Nonword list: mean scores (and SD) for errors identifying each dyslexia type and cutoff values (thresholds according to Crawford and Howell [100], *p* = 0.005) by grade.

Grade	Number of Participants	M (SD) Threshold	Attentional	Letter Position	Multi-letter	Vowel	Consonant	Doubling	Total Errors
2	74	M (SD)	1.6 (1.4)	0.7 (0.8)	1.4 (1.7)	1.8 (1.7)	3.4 (2.4)	2.4 (2.1)	11.2 (6.2)
		threshold	5	3	6	6	10	8	27
3	65	M (SD)	1.1 (1.2)	0.8 (1.0)	0.6 (1.1)	1.1 (1.5)	2.3 (2.0)	1.3 (1.4)	7.4 (4.6)
		threshold	4	4	4	5	8	5	19
4	93	M (SD)	0.9 (1.1)	0.4 (0.6)	0.3 (0.8)	0.9 (1.0)	1.3 (1.5)	0.9 (1.0)	4.7 (3.9)
		threshold	4	2	3	4	5	4	15
5	80	M (SD)	1.0 (1.2)	0.5 (0.8)	0.4 (0.7)	0.6 (0.9)	1.2 (1.4)	0.6 (0.9)	4.2 (3.5)
		threshold	4	3	3	3	5	3	13
6	129	M (SD)	0.7 (0.9)	0.5 (0.8)	0.3 (0.5)	0.7 (0.9)	0.7 (1.0)	0.7 (0.9)	3.5 (3.1)
		threshold	3	3	2	3	4	3	11
7	56	M (SD)	0.5 (0.8)	0.4 (0.7)	0.2 (0.5)	0.6 (0.9)	0.8 (0.9)	0.6 (0.9)	3.2 (3.6)
		threshold	3	3	2	3	3	3	12
8	64	M (SD)	0.5 (0.6)	0.3 (0.5)	0.2 (0.6)	0.6 (1.0)	0.5 (0.6)	0.6 (0.8)	2.7 (2.6)
		threshold	2	2	2	4	2	3	9

**Table 7:** Distribution of the presence/absence of dyslexia according to the *Tiltan-IT* and *Word and Nonword Reading Test* [92].

		*Tiltan-IT*		
		Dyslexia	Typically Developing	Total
*W-NW Reading Test* ^a^	Dyslexia	51	71	122
	Typically developing	59	426	485
	Total	110	497	607

^a^ Eleven recordings of typically developing 5th graders were missed due to technical issues.

**Table 8:** Distribution of the presence/absence of dyslexia according to the *Tiltan-IT* and *MT text reading test* [90,91].

		*Tiltan-IT*		
		Dyslexia	Typically Developing	Total
*MT- text reading test*	Dyslexia	8	7	15
	Typically developing	102	501	603
	Total	110	508	618

## Data Availability

The datasets presented in this article are not readily available because the data are part of an ongoing study. Requests to access the datasets should be directed to the corresponding author.

## Appendix A

**Table A1:** Word list: cutoff values of error scores by grade; percentile and the threshold calculated for Crawford and Howell’s [98] *t*-test, with *p* = 0.005.

Grade	Number of Participants	Thresholds	Attentional	Letter Position	Surface	Multi-letter	Vowel	Consonant	Doubling	Total Errors	Surface Errors in Stress List	Between-Word Migrations in Word Pairs
2	74	10 PR	9	6	10	9	14	11	14	50	8	5
		5 PR	11	8	11	11	17	13	17	57	9	7
		Crawford threshold	15	10	14	16	23	19	24	96	11	8
3	65	10 PR	7	5	8	7	11	8	11	41	7	4
		5 PR	9	7	9	9	14	10	13	47	8	6
		Crawford threshold	12	8	12	9	17	12	16	66	9	8
4	93	10 PR	6	5	7	5	9	6	8	34	6	3
		5 PR	7	6	8	6	11	8	10	39	7	5
		Crawford threshold	8	7	11	7	12	8	9	50	9	5
5	80	10 PR	5	5	7	3	7	4	5	28	5	3
		5 PR	6	6	8	4	9	6	7	32	6	4
		Crawford threshold	7	6	11	5	10	7	7	41	8	4
6	129	10 PR	4	4	6	2	6	3	4	23	5	2
		5 PR	5	5	7	3	7	5	5	27	6	3
		Crawford threshold	5	7	9	3	8	5	6	30	7	3
7	56	10 PR	4	4	6	0	5	2	3	20	4	2
		5 PR	5	5	7	0	6	4	4	23	5	3
		Crawford threshold	6	7	7	3	9	6	6	34	6	3
8	64	10 PR	3	4	4	0	5	2	0	18	4	2
		5 PR	4	5	5	0	6	3	0	21	5	3
		Crawford threshold	6	5	7	3	7	4	5	27	6	3

**Table A2:** Nonword list: cutoff values of error scores by grade; percentile and the threshold calculated for Crawford and Howell’s [98] *t*-test, with *p* = 0.005.

Grade	Number of Participants	Thresholds	Attentional	Letter Position	Multi-letter	Vowel	Consonant	Doubling	Total Errors
2	74	10 PR	3	2	4	4	6	5	14
		5 PR	4	3	5	5	7	6	16
		Crawford threshold	5	3	6	6	10	8	27
3	65	10 PR	3	1	2	3	5	3	12
		5 PR	4	2	3	4	6	4	14
		Crawford threshold	4	4	4	5	8	5	19
4	93	10 PR	2	1	1	2	4	2	10
		5 PR	3	2	2	3	5	3	12
		Crawford threshold	4	2	3	4	5	4	15
5	80	10 PR	2	1	1	2	3	2	9
		5 PR	3	2	2	3	4	3	11
		Crawford threshold	4	3	3	3	5	3	13
6	129	10 PR	2	1	1	2	2	2	8
		5 PR	3	2	2	3	3	3	10
		Crawford threshold	3	3	2	3	4	3	11
7	56	10 PR	2	1	1	2	2	2	8
		5 PR	3	2	2	3	3	3	9
		Crawford threshold	3	3	2	3	3	3	12
8	64	10 PR	1	1	1	1	1	2	6
		5 PR	2	2	2	2	2	3	8
		Crawford threshold	2	2	2	4	2	3	9

**Table A3:** Word + nonword lists: descriptive statistics and cutoff values of error scores by grade; percentile and the threshold calculated for Crawford and Howell’s [98] *t*-test, with *p* = 0.005.

Grade		Attentional	Letter Position	Surface	Multi-letter	Vowel	Consonant	Doubling	Total Errors	Surface Errors in Stress List	Between-Word Migrations in Word Pairs
2	M	6.9	4.2	6.5	5.9	9.8	9.8	10.3	42.6	4.8	2.6
N = 74	SD	4.1	2.8	3.0	5.7	7.1	6.5	7.8	18.6	2.4	2.2
	10 PR	12	7	10	11	17	16	21	64	8	5
	5 PR	14	8	11	14	20	19	26	71	9	7
	Crawford threshold	17	12	14	20	27	26	30	88	11	8
3	M	4.9	3.7	4.3	2.6	6.1	5.1	5.6	28.5	3.1	2.0
N = 65	SD	3.7	2.6	2.9	3.4	5.6	4.6	5.2	15.5	2.1	2.1
	10 PR	9	6	8	8	13	12	13	49	7	4
	5 PR	11	8	9	11	16	15	16	55	8	6
	Crawford threshold	14	10	12	11	20	17	19	66	9	8
4	M	3.2	2.5	4.2	1.7	4.2	3.5	3.4	20.0	3.2	1.3
N = 93	SD	2.8	2.1	2.8	2.4	3.8	3.2	3.2	12.8	2.2	1.5
	10 PR	7	6	7	6	11	9	8	40	6	3
	5 PR	9	7	8	8	13	11	10	45	7	5
	Crawford threshold	10	8	11	8	14	12	11	51	9	5
5	M	3.3	2.3	4.7	1.2	3.9	2.8	2.6	23.7	3.5	0.8
N = 80	SD	2.5	2.1	2.3	1.7	3.2	2.7	2.4	13.5	1.8	1.0
	10 PR	6	5	7	4	8	6	6	34	5	3
	5 PR	8	7	8	6	10	9	8	38	6	4
	Crawford threshold	10	8	11	6	12	10	9	56	8	4
6	M	2.2	2.7	3.5	0.7	2.9	1.9	2.0	16.0	2.8	0.7
N = 129	SD	1.7	2.3	2.1	1.1	2.5	1.8	2.1	8.6	1.7	0.8
	10 PR	5	5	6	2	7	5	5	30	5	2
	5 PR	7	7	7	4	9	7	7	34	6	3
	Crawford threshold	7	9	9	4	9	7	7	37	7	3
7	M	2.5	2.5	2.6	0.8	3.7	2.2	2.6	14.6	2.0	0.6
N = 56	SD	2.1	2.4	1.7	1.1	2.5	2.4	1.9	9.5	1.4	0.8
	10 PR	4	5	6	1	6	4	5	26	4	2
	5 PR	6	7	7	3	8	5	6	29	5	3
	Crawford threshold	8	9	7	4	10	9	8	38	6	3
8	M	2.3	1.7	2.3	0.6	2.8	1.5	1.8	12.5	1.8	0.6
N = 64	SD	1.9	1.5	1.9	1.2	2.4	1.5	1.8	7.2	1.7	0.7
	10 PR	4	5	4	1	6	3	4	20	4	2
	5 PR	5	6	5	3	7	5	5	22	5	3
	Crawford threshold	7	6	7	4	9	6	7	30	6	3

## References

[1] Friedmann N., Coltheart M., Bar-On A., Ravid D. (2018). Types of developmental dyslexia. Handbook of Communication Disorders: Theoretical, Empirical, and Applied Linguistics Perspectives.

[2] Coltheart M., Rastle K., Perry C., Langdon R., Ziegler J. (2001). DRC: A dual Route cascaded model of visual word recognition and reading aloud. Psychol. Rev..

[3] Friedmann N., Gvion A. (2003). TILTAN: A Test Battery for Dyslexias.

[4] Marshall J.C., Malatesha R.N., Whitaker H.A. (1984). Toward a rational taxonomy of the developmental dyslexias. Dyslexia: A Global Issue.

[5] Carr T.H., Davidson B.J., Hawkins H.L. (1978). Perceptual flexibility in word recognition: Strategies affect orthographic computation but not lexical access. J. Exp. Psychol. Hum..

[6] Paap K.R., Noel R.W. (1991). Dual-route models of print to sound: Still a good horse race. Psychol. Res..

[7] Coltheart M. (1981). Disorders of reading and their implications for models of normal reading. Visible Lang..

[8] Ellis A.W. (1993). Reading, Writing, and Dyslexia: A Cognitive Analysis.

[9] Ellis A.W., Flude B.M., Young A.W. (1987). “Neglect dyslexia” and the early visual processing of letters in words and nonwords. Cogn. Neuropsychol..

[10] Ellis A.W., Young A.W. (1988). Human Cognitive Neuropsychology.

[11] Humphreys G.W., Evett L.J., Quinlan P.T. (1990). Orthographic processing in visual word dentification. Cogn. Psychol..

[12] Peressotti F., Grainger J. (1995). Letter position coding in random consonant arrays. Percept. Psychophys..

[13] Brunsdon R., Coltheart M., Nickels L. (2006). Severe developmental letter processing impairment: A treatment case study. Cogn. Neuropsychol..

[14] Friedmann N., Dotan D., Rahamim E. (2010). Is the visual analyzer orthographic-specific? Reading words and numbers in letter position dyslexia. Cortex.

[15] Friedmann N., Gvion A. (2001). Letter position dyslexia. Cogn. Neuropsychol..

[16] Friedmann N., Gvion A., Nisim R. (2015). Insights from developmental and acquired letter position dyslexia on morphological decomposition in reading. Front. Hum. Neurosci..

[17] Friedmann N., Rahamim E. (2007). Developmental letter position dyslexia. J. Neuropsychol..

[18] Friedmann N., Rahamim E. (2014). What can reduce letter migrations in letter position dyslexia?. J. Res. Read..

[19] Keidar R., Friedmann N. (2011). Does Methylphenidate help readers with letter position dyslexia and attentional dyslexia?. Lang. Brain.

[20] Friedmann N., Haddad-Hanna M. (2012). Letter position dyslexia in Arabic: From form to position. Behav. Neurol..

[21] Friedmann N., Haddad-Hanna M., Saiegh-Haddad E., Joshi M. (2014). Types of developmental dyslexia in Arabic. Handbook of Arabic Literacy: Insights and Perspectives.

[22] Kezilas Y., Kohnen S., McKague M., Castles A. (2014). The locus of impairment in English developmental letter position dyslexia. Front. Hum. Neurosci..

[23] Kohnen S., Marinus E., Friedmann N., Anandakumar T., Nickels L., McArthur G., Castles A. (2014). The Letter Position Test (LetPos). http://www.motif.org.au.

[24] Kohnen S., Nickels L., Castles A., Friedmann N., McArthur G. (2012). When ‘slime’ becomes ‘smile’: Developmental letter position dyslexia in English. Neuropsychologia.

[25] Güven S., Friedmann N. (2019). Developmental letter position dyslexia in Turkish, a morphologically rich and orthographically transparent language. Front. Psychol..

[26] Potier Watkins C., Dehaene S., Friedmann N. (2023). Characterizing different types of developmental dyslexias in French: The Malabi Screener. Cogn. Neuropsychol..

[27] Shallice T., Warrington E.K. (1977). The possible role of selective attention in acquired dyslexia. Neuropsychologia.

[28] Friedmann N., Kerbel N., Shvimer L. (2010). Developmental attentional dyslexia. Cortex.

[29] Toledano L., Friedmann N. (2023). Letter migrations between words in reading aloud can result either from an impairment in orthographic input or in phonological output. Brain Sco..

[30] Rayner K., Murphy L.A., Henderson J.M., Pollatsek A. (1989). Selective attentional dyslexia. Cogn. Neuropsychol..

[31] Friedmann N., Nachman-Katz I. (2004). Developmental neglect dyslexia in Hebrew reading child. Cortex.

[32] Nachman-Katz I., Friedmann N. (2007). Developmental neglect dyslexia: Characteristics and directions for treatment. Lang. Brain.

[33] Nachman-Katz I., Friedmann N. (2008). Developmental neglect dyslexia and its effect on number reading. Lang. Brain.

[34] Nachman-Katz I., Friedmann N. (2009). Writing words in developmental neglect dyslexia. Lang. Brain.

[35] Nachman-Katz I., Friedmann N. (2010). An empirical evaluation of treatment directions for developmental neglect dyslexia. Procedia Soc. Behav. Sci..

[36] Marshall J.C., Newcombe F. (1973). Patterns of paralexia: A psycholinguistic approach. J. Psycholinguist. Res..

[37] Morton J., Patterson K., Coltheart M., Patterson K., Marshall J.C. (1980). A new attempt at an interpretation, or an attempt at a new interpretation. Deep Dyslexia.

[38] McCloskey M., Rapp B. (2000). A visually based developmental reading deficit. J. Mem. Lang..

[39] Valdois S., Gérard C., Vanault P., Dugas M. (1995). Peripheral developmental dyslexia: A visual attentional account?. Cogn. Neuropsychol..

[40] Castles A. (1996). Cognitive correlates of developmental surface dyslexia: A single case study. Cogn. Neuropsychol..

[41] Castles A. (2006). The dual route model and the developmental dyslexias. Lond. Rev. Educ..

[42] Castles A., Coltheart M. (1993). Varieties of developmental dyslexia. Cognition.

[43] Castles A., Bates T., Coltheart M. (2006). John Marshall and the developmental dyslexias. Aphasiology.

[44] Coltheart M., Masterson J., Byng S., Prior M., Riddoch J. (1983). Surface Dyslexia. Q. J. Exp. Psychol..

[45] Dulay K.M., Hanley J.R. (2015). Stress errors in a case of developmental surface dyslexia in Filipino. Cogn. Neuropsychol..

[46] Ellis A.W., Lambon Ralph M.A., Morris J., Hunter A., Funnell E. (2000). Surface dyslexia: Description, treatment, and interpretation. Case Studies in the Neuropsychology of Reading.

[47] Ferreres A.R., Cuitino M.M., Olmedo A. (2005). Acquired surface alexia in Spanish: A case report. Behav. Neurol..

[48] Friedmann N., Lukov L. (2008). Developmental surface dyslexias. Cortex.

[49] Hanley J.R., Hastie K., Kay J. (1992). Developmental surface-dyslexia and dysgraphia: An orthographic processing impairment. Q. J. Exp. Psychol. A.

[50] Howard D., Franklin S., Allport A., MacKay D.G., Prinz W. (1987). Three ways for understanding written words, and their use in two contrasting cases of surface-dyslexia (together with an odd routine for making ‘orthographic’ errors in oral word production). Language Perception and Production: Relationships between Listening, Speaking, Reading and Writing.

[51] Kohnen S., Nickels L., Geigis L., Coltheart M., McArthur G., Castles A. (2018). Variations within a subtype: Developmental surface dyslexias in English. Cortex.

[52] Luzzatti C., Stemmer B., Whitaker H.A. (2008). Acquired Reading and Writing Disorders. Handbook of the Neuroscience of Language.

[53] Marinelli C.V., Angelelli P., Notarnicola A., Luzzatti C. (2009). Do Italian dyslexic children use the lexical reading route efficiently? An orthographic judgment task. Read. Writ..

[54] Masterson J., Funnell E. (2000). Developmental surface-dyslexia. Case Studies in the Neuropsychology of Reading.

[55] Newcombe F., Marshall J.C., Patterson K., Marshall J.C., Coltheart M. (1985). Sound-by-sound reading and writing. Surface Dyslexia: Neuropsychological and Cognitive Studies of Phonological Reading.

[56] Ripamonti E., Aggujaro S., Molteni F., Zonca G., Frustaci M., Luzzatti C. (2014). The anatomical foundations of acquired reading disorders: A neuropsychological verification of the dual-route model of reading. Brain Lang..

[57] Sotiropoulos A., Hanley J.R. (2017). Developmental surface and phonological dyslexia in both Greek and English. Cognition.

[58] Spinelli D., Angelelli P., De Luca M., Di Pace E., Judica A., Zoccolotti P. (1997). Developmental surface dyslexia is not associated with deficits in the transient visual system. Neuro Rep..

[59] Temple C.M. (1997). Developmental Cognitive Neuropsychology.

[60] Temple C.M. (1984). Surface-dyslexia in a child with epilepsy. Neuropsychologia.

[61] Tomasino B., Ius T., Skrap M., Luzzatti C. (2020). Phonological and surface dyslexia in individuals with brain tumors: Performance pre-, intra-, immediately post-surgery and at follow-up. Hum. Brain Mapp..

[62] Tomasino B., Marin D., Maieron M., D’Agostini S., Fabbro F., Skrap M., Luzzatti C. (2015). Double-letter processing in surface dyslexia and dysgraphia following a left temporal lesion: A multimodal neuroimaging study. Cortex.

[63] Valdois S., Bosse M.-L., Pellat J. (2003). Phonological and visual processing can dissociate in developmental dyslexia: Evidence from two case studies. Read. Writ..

[64] Weekes B., Coltheart M. (1996). Surface-dyslexia and surface dysgraphia: Treatment studies and their theoretical implications. Cogn. Neuropsychol..

[65] Wybrow D.P., Hanley J.R. (2015). Surface developmental dyslexia is as prevalent as phonological dyslexia when appropriate control groups are employed. Cogn. Neuropsychol..

[66] Zoccolotti P., De Luca M., Di Pace E., Judica A., Orlandi M., Spinelli D. (1999). Markers of developmental surface dyslexia in a language (Italian) with high grapheme–phoneme correspondence. Appl. Psycholinguist..

[67] Temple C.M., Marshall J.C. (1983). A case study of developmental phonological dyslexia. Brit. J. Psychol..

[68] Campbell R., Butterworth B. (1985). Phonological dyslexia and dysgraphia in a highly literate subject: A developmental case with associated deficits of phonemic processing and awareness. Q. J. Exp. Psychol. Hum..

[69] Khentov-Kraus L., Friedmann N. (2011). Dyslexia in vowel letters (DIVL). Lang. Brain.

[70] Traficante D., Luzzatti C., Friedmann N. (2021). A complex view of the grapheme-to-phoneme conversion (GPC) procedure: Evidence for vowel developmental dyslexia from a shallow orthography language. Easy Chair Prepr..

[71] Güven S., Friedmann N. (2021). Vowel dyslexia in Turkish: A window to the complex structure of the sublexical route. PLoS ONE.

[72] Gvion A., Friedmann N. (2010). Dyscravia: Voicing substitution dysgraphia. Neuropsychologia.

[73] Gvion A., Friedmann N. Nasalexia: When *bat xen* becomes *man xed*, and *marak taim* becomes *barak naim*. Proceedings of the 48th Annual Conference of the Israeli Speech Hearing and Language Association.

[74] Dotan D., Friedmann N. (2015). Steps towards understanding the phonological output buffer and its role in the production of numbers, morphemes, and function words. Cortex.

[75] Luzzatti C., Mondini S., Semenza C. (2001). Lexical representation and processing of morphologically complex words: Evidence from the reading performance of an Italian agrammatic patient. Brain Lang..

[76] Johnston R.S. (1983). Developmental deep dyslexia?. Cortex.

[77] Siegel L.S. (1985). Deep dyslexia in childhood?. Brain Lang..

[78] Stuart M., Howard D. (1995). KJ: A developmental deep dyslexia. Cogn. Neuropsychol..

[79] Barbieri E., Aggujaro S., Molteni F., Luzzatti C. (2015). Does argument structure complexity affect reading? A case study of an Italian agrammatic patient with deep dyslexia. Appl. Psycholinguist..

[80] Ciaghi M., Pancheri E., Miceli G. (2010). Semantic Paralexias: A group-case study on the underlying functional mechanisms, incidence and clinical features in a consecutive series of 340 Italian aphasics. Brain Lang..

[81] Coltheart M., Coltheart M., Patterson K., Marshall J.C. (1980). Deep dyslexia: A review of the syndrome. Deep Dyslexia.

[82] Coltheart M., Patterson K., Marshall J., Coltheart M., Patterson K., Marshall J.C. (1987). Deep dyslexia since 1980. Deep Dyslexia.

[83] Marelli M., Aggujaro S., Molteni F., Luzzatti C. (2012). The multiple-lemma representation of Italian compound nouns: A single case study of deep dyslexia. Neuropsychologia.

[84] Marshall J.C., Newcombe F. (1966). Syntactic and semantic errors in paralexia. Neuropsychologia.

[85] Toraldo A., Cattani B., Zonca G., Saletta P., Luzzatti C. (2006). Reading disorders in a language with shallow orthography: A multiple single-case study in Italian. Aphasiology.

[86] AID (Italian Dyslexia Association) (2007). Consensus Conference on Specific Learning Disorders.

[87] American Psychiatric Association (2013). Diagnostic and Statistical Manual of Mental Disorders (DSM-5).

[88] Raven J., Belacchi C., Scalisi T.G., Cannoni E., Cornoldi C. (2008). Coloured Progressive Matrices, It. Ad. CPM.

[89] Raven J. (2008). Standard Progressive Matrices, It. Ad. SPM.

[90] Cornoldi C., Colpo G. (1998). Prove di Lettura MT per la Scuola Elementare—2 [The MT Reading Test for Primary School—2].

[91] Cornoldi C., Colpo G. (1995). Nuove Prove di Lettura MT per la Scuola Media Inferiore [The new MT Reading Test for Middle School].

[92] Zoccolotti P., De Luca M., Di Filippo G., Judica A., Spinelli D. (2005). Prova di Lettura di Parole e Non parole [Word and Nonword Reading Test].

[93] Bertinetto P.M., Burani C., Laudanna A., Marconi L., Ratti D., Rolando C., Thornton A.M. Corpus e Lessico di Frequenza dell’Italiano Scritto (CoLFIS) [Italian Frequency Lexicon of Written Language]. https://linguistica.sns.it/CoLFIS/Home.htm.

[94] Marconi L., Ott M., Pesenti E., Ratti D., Tavella M. (1994). Lessico Elementare: Dati Statistici Sull’Italiano Scritto e Letto dai Bambini delle Elementari [Elementary Lexicon: Statistical Data on Italian Written and Read by Children Attending Primary School].

[95] Mulatti C., Andriolo S. Un Algoritmo per Calcolare la Letter Confusability Media e Totale di Parole e Nonparole. (In Progress). https://lilia.dpss.psy.unipd.it/vicini/vicini2.php.

[96] Shallice T., McGill J., Requin J. (1978). The origins of mixed errors. Attention and Performance VII.

[97] Mozer M.C. (1983). Letter migration in word perception. J. Exp. Psychol. Hum..

[98] McClelland J.L., Mozer M.C. (1986). Perceptual interactions in two word displays: Familiarity and similarity effects. J. Exp. Psychol. Hum..

[99] Temple C.M. (1988). Red is read but eye is blue: A case study of developmental dyslexia and follow-up report. Brain Lang..

[100] Crawford J.R., Howell D.C. (1998). Comparing an individual’s test score against norms derived from small samples. Clin. Neuropsychol..

[101] Lenhard W., Lenhard A., Gary S. (2018). cNORM: Continuous Norming. [R package]. https://CRAN.R-project.org.

[102] (2022). The jamovi Project. jamovi, Version 2.3. [Computer Software]. https://www.jamovi.org.

[103] World Health Organization (2000). The International Classification of Diseases.

[104] Snowling M.J. (2001). From language to reading and dyslexia. Dyslexia.

[105] Tallal P. (1984). Temporal or phonetic processing deficit in dyslexia? That is the question. Appl. Psycholinguist..

[106] Fitch R.H., Miller S., Tallal P. (1997). Neurobiology of speech perception. Annu. Rev. Neurosci..

[107] Stein J. (2001). The magnocellular theory of developmental dyslexia. Dyslexia.

[108] Stein J. (2019). The current status of the magnocellular theory of developmental dyslexia. Neuropsychologia.

[109] Facoetti A., Paganoni P., Turatto M., Marzola V., Mascetti G.G. (2000). Visual-spatial attention in developmental dyslexia. Cortex.

[110] Facoetti A., Lorusso M.L., Paganoni P., Cattaneo C., Galli R., Mascetti G.G. (2003). The time course of attentional focusing in dyslexic and normally reading children. Brain Cogn..

[111] Bouma H., Legein C. (1977). Foveal and parafoveal recognition of letters and words by dyslexics and by average readers. Neuropsychologia.

[112] Pelli D.G., Palomares M., Majaj N.J. (2004). Crowding is unlike ordinary masking: Distinguishing feature integration from detection. J. Vision.

[113] Spinelli D., De Luca M., Judica A., Zoccolotti P. (2002). Crowding effects on word identification in developmental dyslexia. Cortex.

[114] Nicolson R.I., Fawcett A.J. (1990). Automaticity: A new framework for dyslexia research?. Cognition.

[115] Fawcett A.J., Nicolson R.I., Reid G., Fawcett A.J. (2004). Dyslexia: The Role of the Cerebellum. Dyslexia in Context: Research, Policy and Practice.

[116] Cohen L., Lehericy S., Chochon F., Lemer C., Rivaud S., Dehaene S. (2002). Language-specific tuning of visual cortex? Functional properties of the visual word form area. Brain.

[117] Shaywitz S.E., Shaywitz B.A., Pugh K.R., Fulbright R.K., Constable R.T., Mencl W.E., Shankweiler D.P., Liberman A.M., Skudlarski P., Fletcher J.M. (1998). Functional disruption in the organization of the brain for reading in dyslexia. Proc. Natl. Acad. Sci. USA.

[118] Paulesu E., Demonet J.F., Fazio F., McCrory E., Chanoine V., Brunswick N., Cappa S.F., Cossu G., Habib M., Frith C.D. (2001). Dyslexia: Cultural diversity and biological unity. Science.

[119] Richlan F., Kronbichler M., Wimmer H. (2009). Functional abnormalities in the dyslexic brain: A quantitative meta-analysis of neuroimaging studies. Hum. Brain. Mapp..

[120] Paulesu E., Danelli L., Berlingeri M. (2014). Reading the dyslexic brain: Multiple dysfunctional routes revealed by a new meta-analysis of PET and fMRI activation studies. Front. Hum. Neurosci..

